# A Novel Triple-Fluorescent HCMV Strain Reveals Gene Expression Dynamics and Anti-Herpesviral Drug Mechanisms

**DOI:** 10.3389/fcimb.2020.536150

**Published:** 2021-01-08

**Authors:** Ulfert Rand, Tobias Kubsch, Bahram Kasmapour, Luka Cicin-Sain

**Affiliations:** ^1^ Department of Vaccinology and Applied Microbiology, Helmholtz Centre for Infection Research (HZI), Braunschweig, Germany; ^2^ German Centre for Infection Research (DZIF), Hannover-Braunschweig Site, Braunschweig, Germany; ^3^ Centre for Individualised Infection Medicine (CIIM), A Joint Venture of Helmholtz Centre for Infection Research (HZI) and Hannover Medical School (MHH), Braunschweig, Germany; ^4^ Cluster of Excellence RESIST (EXC 2155), Hannover Medical School (MHH), Hannover, Germany

**Keywords:** Human Cytomegalovirus, reporter assay, *in vitro* drug testing, live-cell imaging, herpesvirus, letermovir, antivirals, screening

## Abstract

Human Cytomegalovirus (HCMV) infection may result in severe outcomes in immunocompromised individuals such as AIDS patients, transplant recipients, and neonates. To date, no vaccines are available and there are only few drugs for anti-HCMV therapy. Adverse effects and the continuous emergence of drug-resistance strains require the identification of new drug candidates in the near future. Identification and characterization of such compounds and biological factors requires sensitive and reliable detection techniques of HCMV infection, gene expression and spread. In this work, we present and validate a novel concept for multi-reporter herpesviruses, identified through iterative testing of minimally invasive mutations. We integrated up to three fluorescence reporter genes into replication-competent HCMV strains, generating reporter HCMVs that allow the visualization of replication cycle stages of HCMV, namely the immediate early (IE), early (E), and late (L) phase. Fluorescent proteins with clearly distinguishable emission spectra were linked by 2A peptides to essential viral genes, allowing bicistronic expression of the viral and the fluorescent protein without major effects on viral fitness. By using this triple color reporter HCMV, we monitored gene expression dynamics of the IE, E, and L genes by measuring the fluorescent signal of the viral gene-associated fluorophores within infected cell populations and at high temporal resolution. We demonstrate distinct inhibitory profiles of foscarnet, fomivirsen, phosphonoacetic acid, ganciclovir, and letermovir reflecting their mode-of-action. In conclusion, our data argues that this experimental approach allows the identification and characterization of new drug candidates in a single step.

## Introduction

Cytomegalovirus (CMV) is a common opportunistic infection in immunocompromised hosts or in congenitally infected children (Cannon et al., 2013), where virus replication and cytopathic effects result in tissue damage and disease (Boppana and WJ, 2013). Therefore, several antivirals thwarting virus replication have been approved for clinical use in adult patients (Ljungman et al., 2019).

The pharmacotherapy of CMV infection relies on compounds that specifically target viral replication during the lytic phase, but do not affect cellular processes. They include substances targeting the virus DNA polymerization reaction, such as ganciclovir (Crumpacker, 1996) or foscarnet (Chrisp and Clissold, 1991). Ganciclovir is a guanosine analogue that specifically requires phosphorylation by the CMV-encoded kinase UL97 prior to its incorporation in the DNA. Therefore only in the cells expressing the functional UL97 kinase, ganciclovir may incorporate itself in the growing DNA strand, which slows down its elongation (Biron, 2006). Foscarnet blocks the pyrophosphate binding to the viral DNA polymerase and is closely related to the phosphonoacetic acid (PAA) which is not approved for clinical use. A newly approved antiviral called letermovir targets the DNA terminase complex of cytomegalovirus, impairing viral DNA packaging into capsids (Melendez and Razonable, 2015; Gerna et al., 2019). A third class of antivirals is represented by fomivirsen (ISIS 2922), a single-stranded phosphorothioate oligonucleotide in antisense orientation of the essential immediate-early 2 (IE2) gene. Fomivirsen showed a 30-fold higher potency against CMV *in vitro* than ganciclovir (Azad et al., 1993), which was assumed to depend on its binding to IE2 mRNA (Perry and Balfour, 1999), but later studies noticed that resistance against fomivirsen could be spontaneously acquired in a laboratory HCMV strain that was independent of mutating its target sequence (Mulamba et al., 1998). Therefore, the antiviral activity of fomivirsen might be partially independent of its antisense binding, although its mode of action remains unclear.

Despite the availability of the above-mentioned antivirals in clinics, there are substantial limitations in their use urging for the search for novel anti-CMV compounds. Firstly, none among the available antivirals is approved for the treatment of congenital CMV infection. Secondly, all of the available anti-CMV therapeutics may elicit the emergence of drug-resistance mutations in the virus (Gerna et al., 2019; Griffiths, 2019). Therefore, alternative therapeutic options of CMV disease are needed to close this clinical gap.

The lytic replication cycle of cytomegalovirus divides into three stages. The immediate-early stage initiates as soon as viral DNA reaches the nucleus and starts the transcription of IE genes. The early (E) stage is defined by its dependence on prior expression of viral gene products, which act as transactivators of viral genes expressed in the E stage. Once viral DNA replication initiates, the virus cycle is in the late (L) stage (Stinski, 1978). Importantly, all currently available antivirals target the virus during the L stage of its replication. This could be due to screening assays for the identification of CMV antivirals, which have rested on measuring viral replication in the presence of inhibitors.

Modern approaches to detect antiviral inhibition rely on reporter gene expression systems, where the infection process is discerned quickly and sensitively by means of fluorescent proteins or reporter enzymes, such as luciferases, expressed in transgenic mutant viruses (Ibig-Rehm et al., 2011; Sampaio et al., 2013; Gardner et al., 2015). These approaches allow identifying compounds that impair virus entry into cells or inhibit early stages of the lytic cycle. However, the available approaches have rested on the monitoring of a single infection phase, thus limiting the assay read-out.

Here, we present a novel approach to monitor CMV infection through all its lytic phases. Three reporter genes encoding spectrally different fluorophores were inserted in frame with essential genes of the immediate early, the early, and the late phase of the TB40/BAC4 HCMV strain. We infected cells with the HCMV^3F^ (three fluorescent proteins) virus and treated them with various antivirals as a proof-of-concept. When assessed by live-cell imaging, this revealed distinct dynamic expression profiles of each tagged gene that change characteristically upon treatment. Finally, we demonstrated that flow cytometric analysis complements this information by allowing lytic phase categorization at population level and by revealing changes in the efficiency of viral spread.

## Materials and Methods

### Generation of Recombinant HCMV^3F^

HCMV^3F^ was generated in three rounds of *en passant* mutagenesis (Tischer et al., 2010) on a TB40/BAC4 background (GenBank: EF999921.1) (Sinzger et al., 2008). First, the mNeonGreen gene, encoding a green fluorescent protein (Allele Biotech, USA) (Shaner et al., 2013), linked to the P2A peptide (Kim et al., 2011) was inserted before the start codon of UL122/123 exon 2 as described previously (Kasmapour et al., 2017). Next, the small capsid protein (SCP, UL48A) was labeled with mCherry. As the UL48A ORF overlaps with UL49, mCherry was inserted 12 bp downstream of the start codon. T2A peptide was inserted after mCherry removing its stop codon to maintain the ORF. The start codon and the 12 bps from the start of UL48 were reinserted reconstituting the intact start of UL48A. Lastly, a fusion sequence encoding an SV40-NLS, mTagBFP2 and P2A was introduced at the 5’-end of the E1 (UL112/UL113) coding region leaving the start codon of E1 intact. Cloning design was done with SnapGene software (GSL Biotech, USA). The recombinant BAC was transfected into RPE-1 cells using FuGene HD (Promega, USA) and the reconstituted virus was expanded on HFFF-Tet cells for 10–14 days (Stanton et al., 2010). Virus stocks were generated by removing cell debris through centrifugation (500 × g, 10 min) and pelleting viral particles *via* ultracentrifugation at 24,000 × g, for 4 h at 4°C. This was followed by two steps of homogenization using a Dounce homogenizer and ultracentrifugation in 15% sucrose-containing buffer. HCMV^3F^ was titrated as described before (Kasmapour et al., 2017).

### Cell Culture and Viral Infection

RPE-1 cells (ATCC Cat# CRL-4000, RRID : CVCL_4388) were cultured in DMEM F-12 HAM, supplemented with 5% fetal calf serum (FCS), and 20 nM glutamine. MRC-5 cells (ATCC Cat# CCL-171, RRID : CVCL_0440) were cultured in MEM supplemented with 10% FCS, 20 nM glutamine, and 1 nM sodium pyruvate. Both cell lines were maintained at 37°C, 5% CO_2_, and 100% air humidity. Cells were split twice per week at 1:10. Infection with HCMV^3F^ was done by diluting the virus to the appropriate MOI in fresh cell medium, adding this medium to the vessel and centrifuging the cells for 10 min at room temperature at 800 × g. Then, medium was replenished by fresh, virus-free medium and cells were incubated at 37°C, 5% CO_2_, and 100% air humidity until further experimental procedures.

### Antiviral Treatment

All antiviral reagents, except for fomivirsen, were added immediately following infection. Fomivirsen/ISIS2922 was purchased from Metabion (Planegg, Germany) as fully phosphorothioated oligonucleotide (5’- GCG TTT GCT CTT CTT CTT GCG -3’) and used at 5 µM final concentration, being added to cell culture 1 h before viral infection. Foscarnet (Selleckchem, USA) was used at 200 µM, ganciclovir (Merck, Germany) at 50 µM, phosphonoacetic acid (PAA; Carl Roth, Germany) at 1.8 mM, and letermovir (Cayman Chemicals, USA) at 10 nM final concentration.

### Live-Cell Imaging and Quantification

For live-cell imaging, MRC-5 or RPE-1 cells were seeded at a density of 2 × 10^5^ cells/ml medium onto an eight-well chamber slide (ibidi, Germany). Following viral infection and antiviral treatment, cell nuclei were labeled with SiR-DNA (Spirochrome, Switzerland) at 0.5 µM final concentration. Image acquisition was done using either a Leica SP5 or a Zeiss LSM980 confocal laser-scanning microscope, both equipped with an incubation chamber, maintaining 37°C, 5% CO_2_, and 100% air humidity. Pictures were done every 20–30 min and image stacks were analyzed using ImageJ and custom macros. For single-cell analysis, cell nuclear fluorescence was segmented based on the SiR-DNA signal. Segmentation for population averaging image analysis was done using the mNeonGreen-ie1/2 signal. Virus growth area measurement was done with a Sartorius IncuCyte S3 automated fluorescence microscope. Data was analyzed using Microsoft Excel 2016 (Microsoft, USA) and GraphPad Prism 8 (GraphPad Software, USA).

### RNA-FISH

Custom Stellaris^®^ FISH Probes were designed against HCMV ie1 by utilizing the Stellaris^®^ RNA FISH Probe Designer (Biosearch Technologies, Inc., Petaluma, CA, USA) available online at www.biosearchtech.com/stellarisdesigner (Version 4.2). The HCMV ie1 were hybridized with the Stellaris RNA FISH Probe set labeled with Quasar570 (Biosearch Technologies, Inc.), following the manufacturer’s instructions available online at www.biosearchtech.com/stellarisprotocols.

### Flow Cytometry

For flow cytometric analysis, cells were seeded onto flat bottom 96-well plates (ThermoFisher Scientific, USA) at a density of 2 × 10^5^ cells/ml medium. Before measurements, cells were trypsinized, washed, and resuspended in PBS with 2% FCS. Cell suspensions were then transferred onto a U-bottom 96-well plate (ThermoFisher Scientific, USA) and fluorescence signals were measured in a LSR II (BD Biosciences, USA) equipped with High Throughput Sampler (HTS). Data analysis was done with FlowJo v10 software (Treestar, USA).

## Results

### Integration of Fluorescence Reporter Genes Into the HCMV Genome

**Figure 1:**
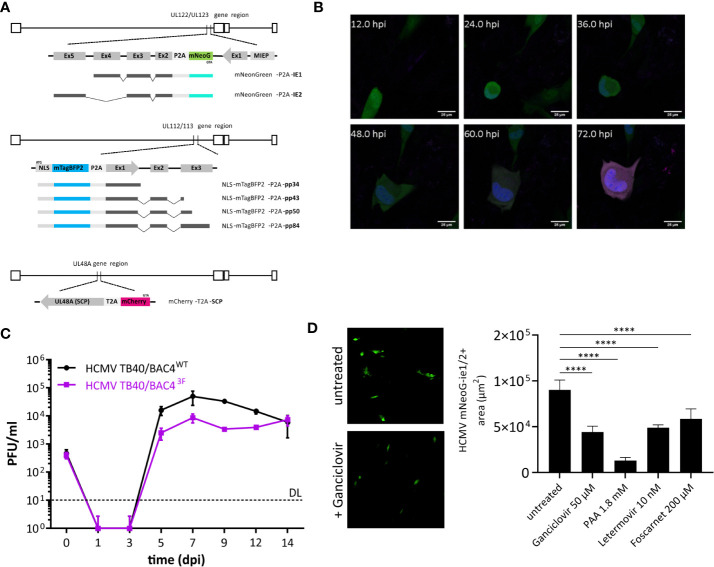
Design and function of a trifluorescent reporter HCMV. **(A)** Novel strain TB40/BAC4 HCMV^3F^ was generated by minimally invasive genetic integrations. Cassettes encoding a fluorescent protein and a 2A peptide were positioned replacing the START codons of UL122/123 (ie1/2), UL112/113 (e1), and SCP (UL48A). **(B)** Expression of mNeonGreen (green), mTagBFP2 (blue), and mCherry (magenta) during the course of primary infection of MRC-5 fibroblasts in live-cell microscopy. **(C)** *In vitro* growth curves of TB40/BAC4 HCMV^3F^ and the parental TB40/BAC4^WT^ virus in MRC-5 fibroblasts. **(D)** Virus growth area measurement. MRC-5 cells were infected with HCMV3F on 96-well plates and mean areas per well showing mNeonGreen-ie1/2 fluorescence were determined microscopically at 5 dpi. (mean values +/− SD; n = 3; PFU, plaque-forming units; DL, detection limit; dpi, days postinfection; D: ****p < 0.0001 with one-way ANOVA and Dunnett’s multiple comparisons test).

Tracking the expression of essential genes allows the identification of antiviral effects by observing a decrease or absence of the reporter signal. Therefore, our reporter virus is convenient for screens of antiviral substances and a rapid identification of compounds that block the virus at a defined stage of its replication cycle. However, modification of CMV genomes to create recombinant reporter strains may lead to losses of viral fitness, especially if endogenous viral promoters driving essential viral genes are used to express reporter genes. Hence, reporter genes driven by ectopic promoters, or those regulating non-essential genes are often used, but they do not accurately reflect viral gene regulation and the impact of antiviral treatment. To bridge this gap, we inserted fluorescence reporter genes directly under control of endogenous HCMV promoters controlling essential viral genes and positioned 2A peptide-encoding sequences that ensure ribosomal skipping (Szymczak and Vignali, 2005) between the reporter gene and the viral gene (**Figure 1A**), allowing co-expression of two separate proteins from a shared transcript. Therefore, the reporter genes in our system accurately reflected viral gene expression while attenuating effects from fusion proteins were prevented.

Experimental methods to sensitively detect blocks and delays in the specific phases of the virus cycle are not available. Therefore, we generated a recombinant virus that expresses reporter genes that represent each of the three lytic phases. Fluorescence protein coding sequence were inserted into the sequence of the TB40/BAC4 strain (Sinzger et al., 1999) maintained as a bacterial artificial chromosome (BAC) in *E. coli*. We used *en passant* mutagenesis, to introduce reporter genes followed in-frame by the 2A peptide sequence directly in front of the start codon of the respective viral gene. These HCMV genes encode pleiotropic factors that are essential during their respective replication cycle phase. Ie1 and ie2 (UL122 and UL123), e1 (UL112/113), and SCP (UL48A) are necessary for transactivation, genome replication, and capsid formation among other important functions, respectively. To minimize the risk of unwanted recombination events, we chose fluorescence genes with low levels of sequence similarity as well as two different 2A peptide sequences (P2A and T2A). In addition, mNeonGreen, mTagBFP2, and mCherry are strictly monomeric proteins. This property prevents multimerization not only of the fluorescence proteins but also of products of occasional read-through, known to occur with 2A peptides (Donnelly et al., 2001). mTagBFP2 tagging the e1 gene was in addition fused with a nuclear localization sequence (NLS) to label nuclei of infected cells. Total ectopic sequences introduced to encode three fluorescence reporters and 2A peptides were 2450 bp, accounting for only ~1% of the viral genome. Altogether, iterative modifications using *en passant* mutagenesis guided a genetic design that came with minimal sequence alterations to create a trifluorescent reporter virus termed HCMV^3F^.

### HCMV^3F^ Represents Lytic Phases of the Virus

Upon infection of naïve human cells, bright fluorescence signals could be observed in live-cell confocal microscopy. Distribution of fluorescence signals also revealed cellular structures, such as the virus assembly compartment as a dim large intracytoplasmic area (**Figure 1B**). Cell-to-cell spread of the virus was visible from 2–3 days postinfection (dpi). However, we observed considerable variability between cells in terms of timing of reporter expression. Therefore, the values stated here represent averages. Altogether, the order of signal appearance within individual cells matched the expected sequence of activation of viral genes during the lytic cycle and is congruent with published data (Stern-Ginossar et al., 2012). Sequence alterations of the CMV genome can alter its growth properties by changing abundance, localization, stability, and/or function of viral factors encoded. In order to assess the level of alteration introduced, we performed a growth assay comparing the recombinant virus with its parental clone TB40/BAC4. MRC-5 human fibroblasts were infected at a multiplicity of infection (MOI) of 0.1 and cultivated for 2 weeks, sampling the supernatant every 2 days (**Figure 1C**). The infectious titer in supernatants was determined by conventional plaque assays. Viral progeny released from infected cells was detected from 5 dpi, reaching peak levels at 7 dpi and remaining at high levels throughout the course of the experiment for both HCMV^3F^ and the parental virus. While HCMV^3F^ produced lower viral titers from 5 to 12 dpi, TB40/BAC4 levels never exceeded those of HCMV^3F^ by more than 10-fold. Given the exponential nature of viral growth as well as the similarity of growth curve shapes between the wild type and HCMV^3F^, we conclude that our recombinant virus can visualize viral dynamics qualitatively while quantitative results have to be considered carefully. In order to determine whether HCMV^3F^ allows the detection of growth inhibition by substances with known antiviral activity, we assessed the expansion of mNeonGreen-ie1/2 fluorescent areas in cell culture (**Figure 1D**). Drugs targeting the replication of viral DNA (foscarnet, ganciclovir, phosphonoacetic acid) or the terminase complex (letermovir) effectively limited cell-to-cell spread of the virus.

In summary, these experiments reveal that HCMV^3F^ visualizes gene expression dynamics in live infected cells of all three lytic phases of CMV replication while remaining sensitive to treatment with anti-herpesviral drugs.

### Live-Cell Analysis Reveals HCMV Dynamic Gene Expression Patterns

**Figure 2:**
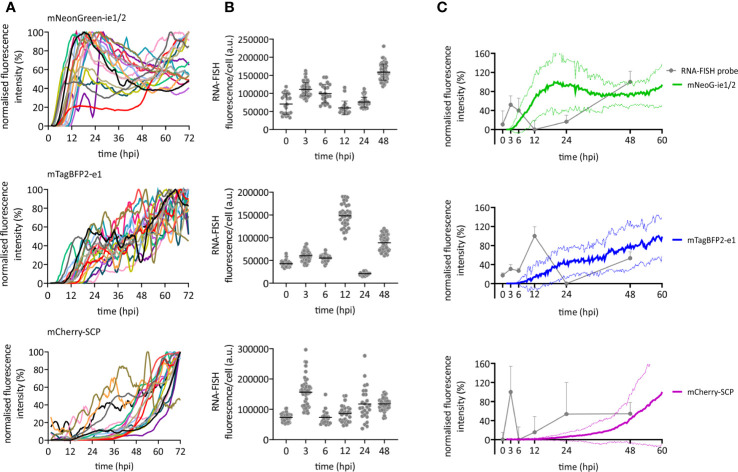
Immediate early, early, and late gene expression dynamics of HCMV. RPE-1 or MRC-5 cells were infected with TB40/BAC4 HCMV^3F^ at MOI 0.5 or MOI 5, respectively, and subjected to live-cell confocal microscopy acquiring images at 20 min intervals. **(A)** Fluorescence profiles of single cells for mNeonGreen-ie1/2, mTagBFP2-e1, and SCP-mCherry [curves were generated by smoothing raw data using the Savitzky-Golay algorithm (10-neighbor), n = 19]. **(B)** RNA-FISH was performed on RPE-1 cells infected with TB40/BAC4 wild type HCMV-infected cells at indicated times postinfection (0 h = not infected). Fluorescence intensity of the fluorescently labeled probe was integrated over whole cell bodies. Each dot represents one cell (n ≥ 20; error bars represent SD). **(C)** Averaged and normalized signals (mean: thick line; SD: dotted line) of fluorescence reporter signals in A (green: mNeonGreen-ie1/2; blue: mTagBFP2-e1; magenta: mCherry-SCP) are compared to averaged and normalized RNA-FISH signals from **(B)**. Error bars and error lines represent SD.

We used our newly developed HCMV^3F^ to dynamically monitor reporter gene expression by confocal live-cell microscopy at high temporal resolution, and thus to monitor the CMV infection dynamics in cell culture over the course of 3 days. We considered two strategies for such monitoring, and their inherent limitations: on the one hand, measuring gene expression by averaging the signal from infected cell populations bears the inherent risk to misinterpret temporal profiles, since they may originate from cell-to-cell heterogeneity or phenotypically distinct subpopulations. On the other hand, single-cell tracking requires fine-tuned image analysis methods and is not an established technique in most labs. Thus, we aimed to identify conditions where averaged signals from microscopic imaging of HCMV^3F^ infected cells resolve infection dynamics in a manner that reflects effects observed at the single cell level. To reduce sources of heterogeneity, we used relatively high multiplicities of infection and synchronized infection through a short (10 min) exposure to virus that was enhanced by centrifugation (Osborn and Walker, 1968). To assess the remaining degree of heterogeneity, we first tracked fluorescence signals from individual cells (**Figure 2A**). mNeonGreen-ie1/2 expression is biphasic in the highly permissive fibroblastic cell line MRC-5. Earliest detectable signals above background started at 4.6 hpi (+/− 3.0 h) [mean +/− standard deviation (SD)], increasing until 19.7 hpi (+/− 13.2 h). mNeonGreen-ie1/2 signals started to increase again at around 36.2 hpi (+/−17.7 h) in most of the cells, which proceeded beyond 72 hpi (**Figure 2A**). Interestingly, the early phase reporter mTagBFP2-e1 did not exhibit this biphasic behavior, but rather showed a continuous increase throughout the experiment, starting at 8.2 hpi (+/− 5 h) (**Figure 2A**). The mCherry-SCP expression as a late gene reporter followed the second phase of the mNeonGreen-ie1/2 signal and started at 48.0 hpi (+/− 15.4 h), although some gene expression was surprisingly detected in some of the cells at early times, before 12 hpi (**Figure 2A**). Late-gene expression is known to require the amplification of viral genomes by the UL54-encoded viral DNA polymerase. mCherry-SCP expression in our model might be regarded as an indirect indicator for this step in lytic HCMV infection. We found that these dynamic gene expression profiles were accurately represented in signal curves averaged over multiple cells (**Figure 2C**, colored lines). While the average signal represents the expected expression pattern, there is clearly considerable variation from the average at the single cell level. Biphasic mNeonGreen-ie1/2 signals, as well as the temporal order of reporter expression was consistent across all cells analyzed, suggesting that under these conditions, phenotypic cell-to-cell variability does not prohibit cell population averaging.

In order to compare the kinetic of HCMV^3F^ reporter signals with wild type HCMV transcripts, we performed fluorescence *in situ* hybridization of mRNAs (RNA-FISH) of those genes that were labeled in HCMV^3F^ (**Figure 2B**). Transcript levels of all three genes essentially recapitulated the patterns seen by monitoring fluorescent reporter gene expression. We observed a temporal delay between RNA-FISH and the reporter protein signal that may be explained by the delays between transcription and translation. In IE1, we noticed the biphasic kinetic of gene expression recapitulated as two distinct peaks of mRNA levels. This early peak was absent from E1, which is known to require IE2 transduction for its gene expression (Malone et al., 1990). and which showed a burst of mRNA levels at 12 hpi. Interestingly, the low level of SCP reporter gene expression at early times was consistent with a surprising initial peak of SCP mRNA at 3 hpi. Taken together, the reporter gene kinetics reflected aggregated mRNA levels observed at distinct times post infection (**Figure 2C**), if one allows for a time delay between transcription and translation.

In summary, temporal profiles of HCMV gene expression across all three phases of lytic replication as reported by HCMV^3F^ are dynamic, specific and rather homogeneous, allowing a highly sensitive read-out in primarily infected cell populations.

### Dynamic Virus Reduction Assay Reveals Specific “Fingerprints” of Antivirals

**Figure 3:**
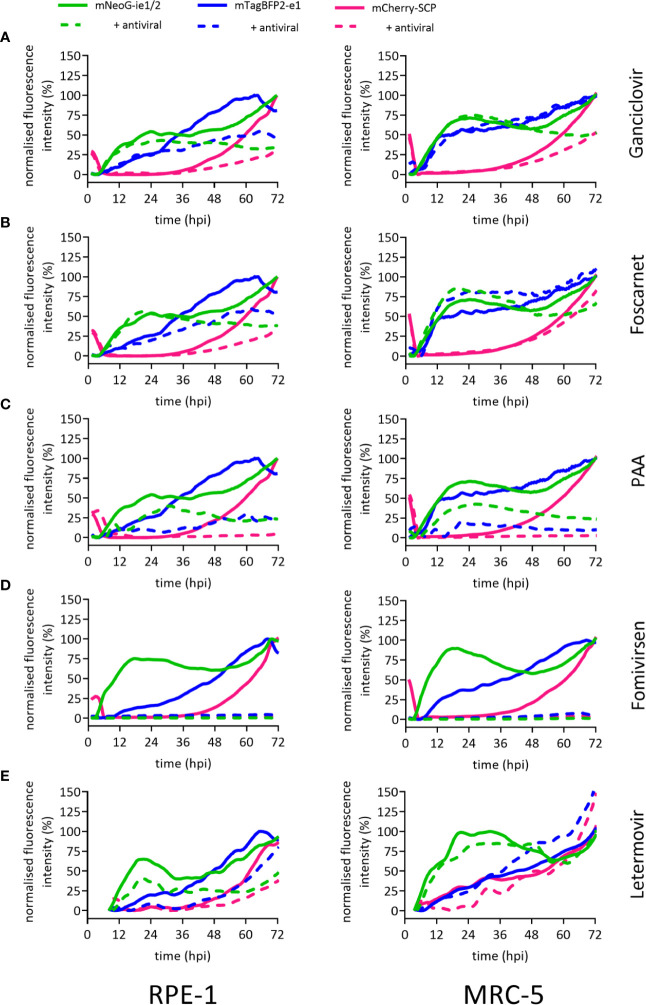
Reporter gene expression dynamics characterize antiviral action of different drugs. Fluorescence signals of RPE-1 or MRC-5 cells infected with HCMV^3F^ at MOI 0.5 or MOI 5, respectively. Cells were either left untreated (solid lines) or treated at the time of infection (except for fomivirsen which was given 1 h before infection) with antiherpesviral drugs (dashed lines) followed by live-cell imaging. Graphs represent mean fluorescence intensity of infected cells within fields of view. Treatment of cells was done with 50 µM ganciclovir **(A)**, 200 µM foscarnet **(B)**, 1.8 mM PAA **(C)**, 5 µM fomivirsen **(D)**, or 10 nM letermovir **(E)**, or 10 nM letermovir. Data represents mean values (n ≥ 3).

The identification of substances and procedures that inhibit viral infection greatly depends on assay sensitivity. To test the applicability of our dynamic virus reduction assay as a tool for the identification of novel antivirals, we assessed our experimental model with well-characterized anti-CMV drugs. These substances covered four different molecular targets. The nucleoside analogue ganciclovir slows DNA elongation when it is incorporated into replicating CMV genomes. At suboptimal *in vitro* dosage, ganciclovir blocked the second expression phase of mNeonGreen-ie1/2 and dampened the mCherry-SCP signal in MRC-5 cells (**Figure 3A**), but did not discernibly affect the first 24 h of infection. The inhibition profile in RPE-1 cells was consistent with that of MRC-5 cells upon ganciclovir treatment (**Figure 3A**). Therefore, the results were in line with a specific antiviral effect in the late phase and affecting the expression of viral genes that depended on replicated viral DNA. Foscarnet, which binds to the pyrophosphate exchange site of the viral DNA polymerase, directly inhibits its enzymatic activity, and thus also blocks viral genome replication. Not surprisingly, the gene expression profile in foscarnet or ganciclovir treated cells was almost identical. Foscarnet at 200 µM concentration, dampened mCherry-SCP expression and the second phase of mNeonGreen-ie1/2 in both tested cell lines (**Figure 3B**). In order to confirm this fingerprint of inhibition, we employed a different pyrophosphate analogue, phosphonoacetic acid (PAA), which is not approved for clinical use. PAA required higher concentrations to be effective against HCMV gene expression. At 1.8 mM, mCherry-SCP and the second phase of mNeonGreen-ie1/2 expression were fully suppressed, arguing for effects in the late phase of the virus cycle. Additionally, a decrease in the mNeonGreen-ie1/2 signal in the immediate early phase activation and in the mTagBFB2-e1 signal were observed, arguing for additional antiviral effects in the ie and e phases of the virus cycle. (**Figure 3C**). This may indicate a less specific activity and potential off-target effects of PAA. Fomivirsen (ISIS2922) is an anti-sense oligonucleotide (ASO) that targets the transcript of the viral ie2 gene, although additional and less specific antiviral effects have been described (Mulamba et al., 1998). In accordance, fomivirsen blocked HCMV gene expression already at the immediate early stage, thus preventing subsequent early and late gene expression (**Figure 3D**). Letermovir, a recently approved “first-in-class” anti-CMV drug, targets the viral terminase complex. HCMV^3F^ reporter expression was minimally altered in MRC-5 cells during the first 72 hpi upon treatment with letermovir (**Figure 3E**). This was consistent with the prediction that letermovir inhibits the virus only after late genes have been expressed and the replicated DNA has been packaged into mature virions. Interestingly, letermovir repressed all three classes of viral genes in RPE-1 cells, which may indicate additional antiviral effects in this cell type. We conclude that our fingerprinting approach to viral gene expression dynamics, based on live-cell imaging of HCMV gene expression in real-time, indicates the mode-of-action of various classes of antiviral drugs, but also may serve to identify hitherto unrecognized antiviral effects. Therefore, it represents a comparably fast (72 h) and sensitive method to identify and classify novel drug candidates.

### Quantification of Infected Population Dynamics Altered by Antivirals

**Figure 4:**
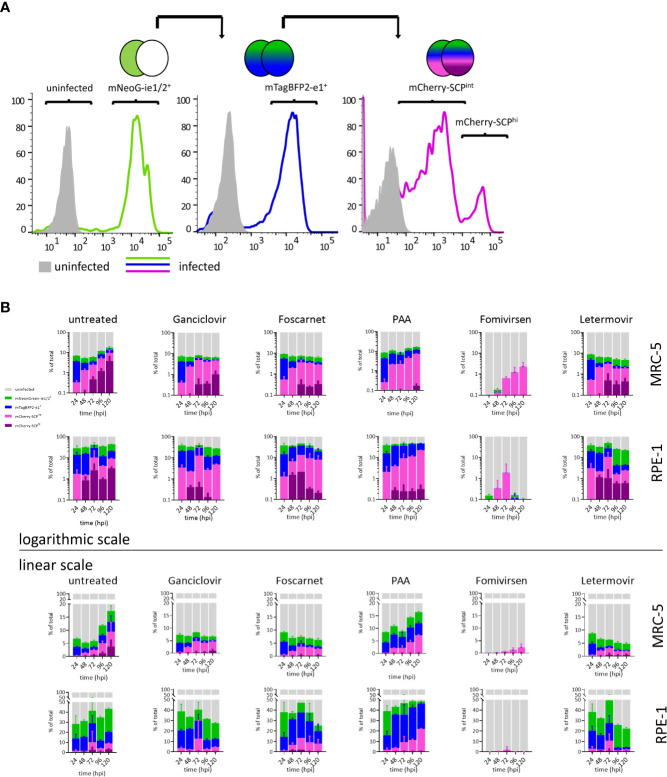
Lytic phase frequency gating of HCMV^3F^-infected cells. MRC-5 or RPE-1 cells were infected with HCMV^3F^ using centrifugal enhancement to achieve 10–20% initially infected cells, respectively. Cells were then either left untreated or treated at the time of infection (except for fomivirsen which was given 1 h before infection) with antiherpesviral drugs and analyzed by flow cytometry at 24 h intervals for 5 days. **(A)** Gating strategy to classify stages of lytic infection in MRC-5 cells. **(B)** Infected MRC-5 and RPE-1 cells were gated into four mutually exclusive lytic stage phases: mNeonGreen-ie1/2^+^, mTagBFP2-e1^+^, SCP-mCherry^int^, and SCP-mCherry^hi^. **(B)** Frequency distribution of lytic stage phases. Representative data from one out of three independent experiments are shown.

Resolving details of antiviral drug action can be done by real-time gene expression fingerprinting of HCMV^3F^-infected cells—without the need for single-cell tracking. However, this method was not suitable to identify drugs that block the release of mature virions and viral cell-to-cell spread. Classically, infectious particles released to the supernatant (or associated with infected cells) are titrated using plaque assays. Incomplete lysis of cells within CMV-associated plaques and the need to incubate supernatants for up to additional 10 days following inoculation make this approach time consuming. In order to test whether the dynamic virus reduction assay with HCMV^3F^ may overcome these limitations, we assessed population frequencies of cells expressing each of the reporter genes by flow cytometry. Progressive gating informed by previous live-cell imaging results (cf. **Figures 2** and **3**) was done to classify i. uninfected, ii. mNeonGreen-ie1/2^+^, iii. mTagBFP2-e1^+^, and, iv. mCherry-SCP^+^ phenotypic subpopulations (**Figure 4A**). mCherry-SCP^+^ cells showed a distinct bimodal distribution after 2–3 dpi, indicating the existence of a “very late” lytic stage. Therefore, we subcategorized cells into mCherry-SCP^int^ and mCherry-SCP^hi^ populations. mCherry-SCP^hi^ cell frequencies were small but showed distinct changes over time. This feature could not be detected in microscopic analysis performed in **Figures 2** and **3**, probably due to the averaging of signals over the cell population. Sampling cells at 24 h intervals for 5 days allowed quantifying primary as well as secondary infection indicated by the increase of infected cells at 96 hpi in MRC-5 fibroblasts and from 72 hpi in RPE-1 cells (**Figure 4B**). We chose to visualize lytic phase frequencies in logarithmic scales to allow representing also small size fraction changes. All antivirals applied prevented cell-to-cell spread of the virus (except for PAA given at suboptimal doses to prevent excessive cytotoxicity). Antiviral effects of all tested drugs were shown by the increased frequency of uninfected cells throughout the experiment. Over the course of the experiment, cell proliferation even exceeded viral proliferation upon ganciclovir, foscarnet, or letermovir treatment, leading to increases in frequencies of uninfected cells over time. Treatment with PAA at suboptimal doses allowed HCMV^3F^ to spread but decreased the frequency of SCP-mCherry^hi^ cells indicating an effect on lytic cycle progression that is still detectable. Fomivirsen, in line with our results depicted in **Figure 3**, almost entirely prevented infection with no more than 2% of cells escaping this protective effect. Letermovir did not interfere with the lytic cycle progression of HCMV. Its effects were visible as continuously decreasing rates of infected cells consistent with its specific interference with the viral terminase complex. In summary, these results show that quantifying phenotypic subpopulation frequencies of HCMV^3F^ infected cells can complement the identification and characterization of antivirals, especially in terms of their effects on viral cell-to-cell spread.

## Discussion

Contemporary advances in transcriptome analysis have exposed a surprising complexity of CMV gene expression over time (Marcinowski et al., 2012; Stern-Ginossar et al., 2012; Weekes et al., 2014; Shnayder et al., 2018; Erhard et al., 2019). The pattern of the viral gene expression varies not only between different cell lines (Towler et al., 2012), but also at the single cell level (Erhard et al., 2019; Shnayder et al., 2020). This implies that no single viral gene can be a reliable proxy of CMV infection in all settings. We reasoned that an accurate assessment of the infection process requires simultaneous monitoring the expression of multiple viral genes. Any such system would require that the virus replication matches the real-life conditions and that the reporter genes are driven by endogenous promoters driving the expression of essential viral genes.

Here, we show a novel concept for antiviral compound characterization in a single assay, covering the expression of essential genes from all three phases of the CMV lytic cycle. We aimed at overcoming disadvantages of previous approaches, which focused on single steps of the viral infection, like entry (Sampaio et al., 2013), or allowed only one-dimensional screening (Gardner et al., 2015; Aryal et al., 2019), required the genetic modification of the host cell (McCormick et al., 2018), or lack temporal resolution (Khan et al., 2017). Shortcomings of existing experimental strategies to screen for antivirals were overcome by the genetic design of the recombinant HCMV^3F^ reporter virus: three fluorescence proteins report the activation of essential viral genes in the same cell (**Figure 1**). This setup revealed distinct dynamic profiles of the ie1/2, e1, and SCP genes over the course of 3–5 dpi (**Figures 2** and **3**) that were consistent with published transcriptomic data (Stern-Ginossar et al., 2012). A unique feature of this system is that it allows combining drug identification and preliminary characterization in a single step. The advantage of this approach is to rapidly identify drugs that act at early stages of the cycle, before genome replication can occur. This means that the virus replication is inhibited by targeting the genetic product of the incoming virus genome, instead of gene products from a population of replicating genomes in the same cells, which should reduce the occurrence of escape mutants. We compared mNeonGreen-P2A-ie1/2 signal dynamics with ie1 mRNA levels at the single-cell level and found a decrease of transcript levels between 3 and 24 hpi that is not reflected at reporter level, probably due to the stability of the fluorescence protein (**Figure 2C**). Using a destabilized reporter protein could enable to follow transcript dynamics more accurately and reflect better the expression of the short-lived ie2 compared to the rather stable ie1 protein alongside other differential regulations of the two splice variants [reviewed in (Adamson and Nevels, 2020)]. The gene expression profiles were rather conserved in the two cell types that we tested, but were characteristically bent by the treatment with different antivirals. To test in detail how HCMV infection changes in the presence of specific antiviral compounds, we designed two complementary experimental strategies. Firstly, live-cell imaging clearly distinguished between antivirals acting at the level of viral DNA synthesis (ganciclovir, PAA, and foscarnet) and immediate early transcripts (fomivirsen). Secondly, flow cytometric measurements assessed secondary infection events, identifying the effect of drugs targeting very late stages of the cycle, including one that takes effects only at the stage of releasing mature virions from the infected cell (letermovir) (**Figure 4**). It is important to note that the two systems provide complementary evidence on the virus replication cycle, where the live cell imaging data showed the overall expression levels of the reporter gene, whereas flow-cytometry revealed the frequency of cells in the stage of the replication cycle represented by the same reporter. Hence, the results were mostly, but not always overlapping, as for instance was the case in cells treated with ganciclovir, where imaging (**Figure 3**) showed a more pronounced effect, while flow-cytometry merely showed a reduction of cells in the gate with high fluorescent indices of the late reporter gene (**Figure 4**). This however would be consistent with ganciclovir effects on late gene expression from replicating genomes, where the second peak of the IE gene and the large expansion of scp are thwarted (**Figure 3A**) by a drug that blocks DNA polymerase activity. Finally, one has to note that we provided antivirals only at the time of infection, and thus the effects of antiviral drugs that target immediate early genes were more pronounced than those acting on the late genes. This might be due to half-life issues of the late acting drugs, so that they might have in part decayed at stages when the virus was reaching the late stage of its replication cycle. A pending question, is how to identify drug candidates for pre-emptive therapy, which would target HCMV latent infection. In contrast to lytic infection, the virus’ gene expression during latency is less well understood, and recent research conflicts about the presence or absence of a latency-associated transcriptional program (Cheng et al., 2017; Shnayder et al., 2018). However, our approach may contribute to the clarification of this question, because the development of reporter viruses driven by the UL138 promoter may provide clear answers on its dynamic of gene expression in latently infected cells and may be addressed in future research. Our approach lays the foundation for such efforts and opens the door to a better understanding of viral transcriptional regulation and replication.

## Data Availability Statement

The original contributions presented in the study are included in the article, further inquiries can be directed to the corresponding author.

## Author Contributions

TK, UR, and BK conducted experiments. UR and LC-S wrote the manuscript. All authors contributed to the article and approved the submitted version.

## Funding

This project was funded by the Deutsche Forschungsgemeinschaft (DFG, German Research Foundation) – Projektnummer 158989968 - SFB 900, by the German Federal Ministry of Science and Education through the project number 031L0005A (Infect-ERANet eDEVILLI) and by the European Research Council through the ERC-PoC project VIVAVE 737592 to LC-S.

## Conflict of Interest

The authors declare that the research was conducted in the absence of any commercial or financial relationships that could be construed as a potential conflict of interest.
